# Entraining IDyOT: Timing in the Information Dynamics of Thinking

**DOI:** 10.3389/fpsyg.2016.01575

**Published:** 2016-10-18

**Authors:** Jamie Forth, Kat Agres, Matthew Purver, Geraint A. Wiggins

**Affiliations:** Computational Creativity Lab, Computational Linguistics Lab, Cognitive Science Group, School of Electronic Engineering and Computer Science, Queen Mary University of LondonLondon, UK

**Keywords:** rhythm, entrainment, cognition, information dynamics, cognitive modeling

## Abstract

We present a novel hypothetical account of entrainment in music and language, in context of the Information Dynamics of Thinking model, IDyOT. The extended model affords an alternative view of entrainment, and its companion term, pulse, from earlier accounts. The model is based on hierarchical, statistical prediction, modeling expectations of both what an event will be and when it will happen. As such, it constitutes a kind of predictive coding, with a particular novel hypothetical implementation. Here, we focus on the model's mechanism for predicting when a perceptual event will happen, given an existing sequence of past events, which may be musical or linguistic. We propose a range of tests to validate or falsify the model, at various different levels of abstraction, and argue that computational modeling in general, and this model in particular, can offer a means of providing limited but useful evidence for evolutionary hypotheses.

## 1. Introduction

We propose a hypothetical anticipatory model of the perception and cognition of events in time. A model of sequence learning and generation from statistical linguistics has been adapted to handle the strongly multidimensional aspects of music, including musical time (Gabrielsson, 1973a,b; Jones, 1976, 1981; Conklin and Witten, 1995; Pearce, 2005; Pearce et al., 2005). Multidimensionality is a property also of language that can usefully be captured The model is called IDyOT, (Information Dynamics of Thinking). IDyOT is a cognitive architecture, after Baars' (1988) Global Workspace Theory: the aim is to capture as much as possible of the framework of basic cognitive function in one uniform processing cycle.

We approach the perception and cognition of musical and lingustic timing from two perspectives. Firstly, in the context of music, we discuss a conceptual space (Gärdenfors, 2000) representation of metrical time (Forth, 2012). The approach enables precise specification of metrical structures, hypothesized as patterns of entrainment that guide attention in musical listening (London, 2012). This perspective can be understood as a top-down specification of a theoretical notion of meter. Our second perspective is bottom-up: a mechanism that we hypothesize is capable of learning such a hierarchical representation of metrical time from exposure to the statistical regularity inherent in music and everyday perceptual experience. Our argument is that musical listening is coordinated by attentional patterns, which arise from a process involving both endogenous generation and induction from perceptual information. Furthermore, we argue that the same process underlies the temporal regulation of cognition in general, and we consider evidence from the domain of natural language to substantiate this claim.

IDyOT computes anticipatory distributions at multiple levels of granularity with respect to the surface sequence, and we hypothesize that the requirement for the temporal predictions of the different levels to coincide is what creates the human tendency toward cyclic (even if non-isochronous) meters in music and poetry. Thus, the work presents a new perspective on the debate between oscillatory and timer-based models (Hass and Durstewitz, 2014). Further, we propose that the combination of coinciding expectations at different levels of granularity are responsible for the percept of meter, explaining the effect modeled by London's (2012) additive cycle approach to metrical strength.

## 2. Theory and hypotheses

Since IDyOT is a multi-faceted theory, we must decompose it. Introductory descriptions are given by Wiggins (2012b) and Wiggins and Forth (2015), and summarized below. Here, we list our specific hypotheses, to map out the subsequent argument.

- We begin from the hypothesis, familiar in cognitive science, that the brain/nervous system is an information processing organ, embodied or in isolation. Specifically, our perspective is that the brain/nervous system is a *statistical* information processing system. More specifically still, our perspective is that the brain/nervous system is a *sequential predictor*: not only does it serve the function of quasi-probabilistically deducing the sources and nature of stimulus received, but it also serves to predict future events in the world. This *sequential* prediction element differs from statistical models of cognition, whose primary concern is deducing the likely cause of current input.
- We propose a particular perceptual learning mechanism, related to but different from extant others, and conceived as a cognitive architecture. It constructs a simulated, hierarchical mental model of the perceptual history of an organism, from which predictions about future states of the world, at multiple levels of abstraction, can be generated. This, when implemented, constitutes a testable hypothesis: its behavior can be compared with extant humans and thus, it may be falsified. Here, we focus on the temporal aspects of the model; falsification in these terms might entail falsification of just the temporal part of the model, or of its entirety, depending on the exact outcomes.
- The underlying principle of the model is that of *information efficiency*: everything the model does is aimed at reducing the computational expense of processing the information to which it is exposed. Thus, there are measures to optimize both the storage and representation of information, and the use of information in predicting future states of the world. We suggest that this is a principle that is likely to hold in biology, because cognitive substrate (nervous tissue) is very expensive to grow and operate, and therefore that there is pressure to optimize its utility. Evidence for this approach, in terms of modeling current human behavior, is given by Pearce (2005) and Pearce and Wiggins (2012).
- Our proposal offers a hypothetical account of the relationship between cortical volume and mind function, including temporal memory, and of why humans entrain in ways not reliably observed in other species. This hypothesis may also be tested by comparison with extant humans coupled with analysis of brain volumes in other mammalian species, and with evidence from the fossil record, using the methodology in Section 5.
- Finally, the methodology espoused in Section 5 may be applied to any aspect of the model with support from empirical study of current biology, to hypothesize about evolution, in two ways: first, the relationships between known developments in species (e.g., cortical volume) and parameters of the model may be investigated; and, second, differently parameterized versions of the model may be allowed to compete in a simulated environment, testing the evolutionary value of its various features.

In the following sections, we lay out the details of our motivation and of the temporal aspects of IDyOT.

## 3. Rhythm and timing in sequential perception

### 3.1. Prediction in temporal perception: concepts and terminology

The key idea of IDyOT is that one route to evolutionary success is for an organism to predict what is likely to happen next in its environment, and that the ability to learn an appropriate model of experience to inform such predictions is an important cognitive ability of higher animals. Further, we propose that the value of such prediction is increased if the prediction of *what* is to happen is coupled with the prediction of *when* it will happen. Playing music, alongside many survival traits, requires the ability to judge precisely where in time an action should be placed, usually anticipating the exact moment with motor preparation so that timing of sound and/or movement is correlated with other activity in the world. It is self-evident that organisms without human-scale cortical development are capable of impressive feats of prediction coupled with synchronization: for example, chameleons catching fast-flying insects, and dogs catching balls; what is not evident in these organisms is the *voluntary maintenance and repetition of such behaviors* in rhythmic synchronization with external stimuli.

Fitch (2013) surveys usages of terms relating to general and musical timing. Our taxonomy describes the same broad phenomena, but is different, and we must clarify our usage, and how it differs from Fitch's. Fitch argues that timing is an example of hierarchical cognition, and we agree. However, as will become clear later, in our model, concepts such as *pulse, meter*, while certainly hierarchical, are explicated in terms of the underlying predictive mechanism, and do not require separate explanations of their own. Particularly problematic are Fitch's notions of *pulse* and *entrainment*. Pulse is introduced thus:

First, rhythmic cognition typically involves extracting a pulseİ or tactusİ at a particular rate (the tempo) that serves as a basis for organizing and structuring incoming sonic events.                             (Fitch, 2013, p. 2)

and, later,

An important characteristic of musical rhythm, …, is isochronicity.                             (Fitch, 2013, p. 2)

Fitch (2013) also notes, later, that it is not the case that all musics display a(n isochronous) pulse—South Asian and Middle Eastern musics often do not have a pulse in this simplified Western sense. This is problematic, because (in the absence of a stated alternative) it implies that these musics have no “basis for organizing and structuring incoming sonic events.” We believe a different definition is required.

For us, *entrainment* is the key concept. Fitch's definition runs thus, in terms of his notion of pulse:

When listeners extract a pulse from the acoustic surface, and adjust their own behavior to it (whether their own acoustic output, in ensemble playing, or their movements, as in dance) this is called **entrainment**.                             (Fitch, 2013, p. 3)

In this definition, entrainment is dependent on the *presence* of a pulse, that is *extracted* and that is, by definition, isochronous. Therefore, the movement of Indian physical performers that is correlated with their culture's non-isochronous music is not entrained. Evidence suggests that this is a narrow definition (Clayton, 2007).

The problem arises from the Western-centric notion that the perception of pulse *precedes* rhythm and meter perception. We suggest that the experience of pulse (isochronous or otherwise) is not primary, but an epiphenomenon of the statistical structure of music. Therefore, we define entrainment differently, allowing our Indian dancers to be entrained:

*Entrainment* is the capacity to sustainedly synchronize with the placement of extrinsic patterns of events in time. *Autonomic* entrainment is the capacity of an organism to entrain without intentional involvement (e.g., in fireflies); this is the kind of entrainment that cannot be switched off by the organism exhibiting it. *Voluntary* entrainment is the capacity of an organism to entrain at a non-autonomic level (e.g., Snowball the cockatoo: Patel et al., 2009; Schachner et al., 2009). *Sustained* voluntary entrainment is the capacity of an organism to entrain non-autonomically without extrinsic encouragement or reward (humans are the only known example).

Musical entrainment can be extremely complicated, with irregular rhythmic structures spanning cycles of several seconds, (e.g., Greek folk music), or with simultaneous multiple levels of synchronization at different speeds and with very subtle deviations from a relatively simple regular beat which are highly musically salient, (e.g., funk and rap). Equally complicated, though different, entrainment is required for production and comprehension of speech. It follows that entrainment is extremely advanced in humans, even though it needs individual development to reach the degrees of hierarchy and precision found in musicians and dancers. Given that such rhythmic sophistication is hard to motivate from a purely biological evolutionary perspective (see e.g., Merchant and Honing, 2014; Merchant et al., 2015, for discussion of such biological evolution), either it must arise *de novo* from social evolutionary pressures; or from a mechanism capable of capturing the simpler rhythms experienced in the world, which is then able to construct complexity as needed; or perhaps a combination of the two (Bown, 2008; Bown and Wiggins, 2009; Merker et al., 2009; Fitch, 2012; Bowling et al., 2013; Ravignani et al., 2013).

We propose that the last of these options is the case: a bottom-up hierarchical perceptual construction of temporal sequence accounts for rhythm and meter in music and language. It has been selected for because it promotes predictive power which enhances information processing and the action that results from it. The enhancement is achieved by *attentional orientation*, which we discuss next.

### 3.2. Attentional orientation

The orientation of attention toward specific spatial locations, objects or moments in time to optimize behavior has been extensively investigated. Coull et al. (2000) describe two distinct forms of attentional shift: *endogenous*, a top-down mechanism initiated to meet cognitive demands, and *exogenous*, a bottom-up mechanism stimulated by unexpected events.

Cherry (1953) investigated auditory selective attention (the “cocktail party effect”) in experiments designed to reveal the extent to which, and under what conditions, listeners could disambiguate simultaneously spoken, but spatially-separated, dialogues recorded by the same speaker. Wearing headphones, subjects were asked to attend only to the speech signal delivered to their right ear and to repeat the words while doing so. Subjects could reproduce the spoken dialogue perfectly, and when subsequently questioned, were largely unable to report any detail from the unattended source, beyond general characteristic such as speech vs. non-speech, and male vs. female speaker. However, in a subsequent experiment, subjects performed the same task but with stimuli consisting of a single speech signal delivered independently to each ear with decreasing inter-ear time delay. In this case, nearly all subjects reported that they recognized that the two signals were the same when the delay was in the region of 2–6 s, suggesting that unattended signals are processed to some degree, and under certain conditions, are able to impact on conscious awareness. This behavior is a necessary consequence of the IDyOT architecture.

Cherry concluded that this mechanism was statistical in nature, and that the brain stored transition probabilities, to be able to estimate maximum-likelihood to guide perception and overcome noisy signals. This was assumed to account for the fact that even dialogues spoken by the same speaker, presented simultaneously but non-spatially separated, could eventually be disambiguated after multiple hearings (up to 10–20 times). Further evidence is provided by a variant of the previous experiment involving the recognition of cliché phrases. The dialogues consisted entirely of concatenated cliché phrases. Participants were reliably able to detect whole phrases at a time with relative ease, presumably relying on highly likely word transitions inherent to cliché phrases. However, between phrases, no expectations could be generated, and participants were equally likely to switch between dialogues at such phrase boundaries, and were therefore unable to completely disambiguate the two dialogues. The IDyOT prediction mechanism accords with this.

In addition to spatial information, listeners can also use other stimulus features, such as pitch, to orientate attention toward particular events (Woods et al., 1991; Woods and Alain, 1993). Semantic salience impacts selective attention, and top-down attention is also mediated by location, pitch, timbre, intensity (Shinn-Cunningham, 2008).

In the visual modality, Posner et al. (1980) developed a reaction time paradigm to provide evidence in support of a theoretical attentional framework consisting of a limited-capacity attentional mechanism coupled with adaptive expectation of where signals were likely to appear in the visual field.

Crucial to the temporal aspect of IDyOT developed below, time itself is also a modulatory factor for attentional orientation. Coull (2004, p. 217) distinguishes between temporal attentional orienting (‘how attentional processing varies as a function of time’) and temporal selective attention (‘how time perception varies as a function of attentional selectivity’).

ERP evidence demonstrates that sounds presented at attended times elicit a larger N1 than sounds at unattended times (Lange et al., 2003; Sanders and Astheimer, 2008). direct comparison between the neural correlates of spatial vs. temporal cues, revealing that both temporal cues (when a target will appear) and spatial cues (where it will appear) similarly improve reaction time, but that hemispheric asymmetry is evident between the two conditions. Similar findings are also reported by Nobre (2001) and Griffin (2002). Nobre (2001, p. 1320) demonstrates that there is no hardwired cue interval, but that “the utility of a warning cue depends upon the specific temporal information it carries and the degree of certainty.” A hypothesized relationship between temporal uncertainty and attentional focus has long been the subject of empirical investigation. Early work by Klemmer (1956, 1957) proposed a model of the relationship between reaction time and an information-theoretic measure of time uncertainty. In processing language, preschool children and adults employ temporally selective attention to preferentially process the initial portions of words in continuous speech. Doing so is an effective listening strategy since word-initial segments are highly informative (Astheimer and Sanders, 2009, 2012).

### 3.3. Entrainment

Our argument, then, is that patterns of events in the world afford entrainment, which in turn affords attention-orienting behavior, if there is a perceptible regularity to the patterns' occurrence, across a range of time-scales. Regularity and periodicity are therefore invariant qualities in perception over time, a fact which sits neatly with the general principle that sequential grouping of events enhances prediction and leverage and/or understanding of causality. In music, the notion of perceptual invariance is reflected in the language used to describe highly periodic rhythms, which are sometimes referred to as *stationary* (Shmulevich and Povel, 2000). More generally, the occurrence of invariance in the natural world is highly suggestive of intentional behavior, such as the distinctive footfalls of a predator or chosen mate. Furthermore, an argument for the evolutionary adaptive quality of entrainment can be made in terms of social interaction and cohesion. To interact and to co-operate successfully in the world, humans must be able to synchronize movement. Synchronization requires accurate temporal prediction to engage the necessary motor control prior to an anticipated timepoint: successful co-ordination cannot be based on reactivity (Trevarthen, 1999–2000; Clayton et al., 2004). Crucially, our perception of temporal invariance and capacity for entrainment allow us to direct attentional resources toward probably-salient moments of time; thus, we better predict events in the world and accordingly act more efficiently. Efficiency, in this context, is a survival trait.

Entrainment capacity in non-humans has been supposed to correlate with the capacity for vocal learning (Patel, 2006; Schachner et al., 2009), though this is now contested (Wilson and Cook, 2016). Even so, from this, and other evidence from lingustics, it may be that entrainment is related to the process of vocal imitation. This, in turn, is implicated in learning to speak (Speidel and Nelson, 2012), which entails speech perception (even prior to the development of semantic association and of speech production). A reason for entrainment to be related to all these things would be cognitive efficiency, according with the underlying principle in IDyOT. Attending to speech, as to anything else, is energetically expensive. If periods of attention can be appropriately timed, by predicting when the next unit of information from an interlocutor will appear, such as orienting attention toward initial portions of words in continuous speech (Astheimer and Sanders, 2012), the efficiency of attending is optimized (Large and Jones, 1999). Further, it is easy to imagine situations where the capacity for physical synchronization would be of survival benefit to early humans: for example, the ability to walk in step, but with irregular paces, to minimize the audible traces of a hunting party. Further, shared entrainment would be a necessary feature of effective sustained conversation, because synchronized prediction in a listener greatly increases the likelihood of successful information transmission. Models of musical and language entrainment are similar, though language seems to be more tolerant of expectation breach: an equally hierarchical system of *beats* for linguistic synchronization is a given in phonology (e.g., Hawkins and Smith, 2001; Hawkins, 2003).

Humans can entrain to a beat, even when it is irregular or variable, and many find it difficult *not* to do so, when presented with music that they find engaging. The phenomenon is studied extensively in the music cognition literature, along with timing and rhythm (e.g., Patel and Daniele, 2003; Cross and Woodruff, 2008; Cross, 2009; Repp, 2011; Fitch, 2012, 2013; London, 2012; Merchant et al., 2015). Some non-human species exhibit temporary entrainment to music when encouraged to do so (Patel et al., 2009; Schachner et al., 2009), and others, such as crickets, exhibit synchronization via reflex response (e.g., Hartbauer et al., 2005), but sustained self-motivated active entrainment seems to be unique to humans (Wilson and Cook, 2016). Grahn (2012) gives a useful survey of related research in neuroscience.

The question of whether such control is achieved by oscillators or by interval timers remains open: Grahn (2012) presents evidence for timer-based control, while Large and Jones (1999) argue for oscillators. See Hass and Durstewitz (2014) for a wider survey of contending models. Evidence from music, beyond the Western tendency toward regular binary or ternary divisions, clearly undermines a naïve oscillator model in which phase-locked oscillators simply oscillate to determine meter: otherwise, fairly simple, naturally divisible meters such as ${}_{8}^{7}$ and the jazz favorite ${}_{4}^{3}{+}_{8}^{3}$ would be at best problematic, and the long cycles of groupings of irregular length found in Greek, Arabic and Indian music would be inexplicable. Because our model operates at a fairly high level of abstraction from the neurophysiology, it is relevant to note that an oscillator can be implemented as a timer, repeatedly triggered. Thus, at more abstract levels of modeling, the distinction is only semantic, and the effect can reasonably be simulated without addressing the detail of the neural implementation. Then, a given temporal interval may be represented by a parameter, forming a closed system with the oscillator or timer that accepts it.

An important class of approaches to these issues lies in the literature on Predictive Coding (e.g., Friston, 2010) and Bayesian Inference (e.g., Tenenbaum et al., 2011). These approaches have been investigated on the neuroscientific level by, for example, Vuust et al. (2009), Vuust and Witek (2014), Vuust et al. (2014), and Honing et al. (2014). Vuust et al.'s work, in particular, presents neuroscientific evidence for a theoretical model with a similar motivation to that presented here. As such, the present work may offer a more detailed explanatory account of the observed neurophysiological responses, as suggested by Maloney and Mamassian (2009) and Wiggins (2011).

Next, we discuss rhythm and meter in language and music, and the affective effects of expectation in pitch and rhythm, in context of our definition of entrainment. There has been further debate elsewhere over the relationship in the literature (e.g., Patel, 2008; Jackendoff, 2009; Fabb and Halle, 2012), which there is not space to survey here.

### 3.4. Rhythm and meter in language

Speech naturally shows regularities in timing, their nature varying across languages. Until recently the view was that these rhythmic differences stem from *isochrony*—an even distribution of certain segment types over time—with individual languages either syllable-timed, mora-timed or stress-timed (e.g., Abercrombie, 1967). For example, whereas Italian speakers appear to maintain approximately equal durations for each syllable, English speakers tend to adjust their speech rate to maintain approximately equal durations between stressed syllables, even when multiple unstressed syllables are interposed:

(1) 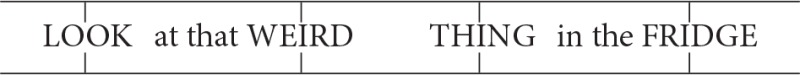


However, empirical evidence does not uphold this strict typological division, with some languages falling somewhere between syllable- and stress-timed (e.g., Dimitrova, 1997). Instead, research suggests that all languages are effectively stress-timed and that the apparent typological differences can be accounted for via differences in stress prominence, syllable complexity, and variability of duration of vowels and consonants (Dauer, 1983; Grabe and Low, 2003; Patel and Daniele, 2003; Patel, 2008). These differences lead to the impression of different rhythmic classes and perhaps, via their effects on perception and predictability, to the segmentation unit naturally used by speakers and acquired by infants (Nespor et al., 2011). Indeed, psycholinguistic evidence shows that rhythm and timing play a role in perception, with rhythmic stress affecting attention given to phonemes (Pitt and Samuel, 1990), expectations set up by syllable stress or intonation patterns early in a sentence affecting the perceived identity of ambiguous words later on (Dilley and McAuley, 2008), and regularity in timing speeding up processing (Quené and Port, 2005). Effects are also seen in production: even infant babbling shows syllable timing patterns characteristic of the language being learned (Levitt and Wang, 1991).

Rhythm and timing are, of course, not fixed, and here expectation and predictability play a significant role. Information content has effects both globally, with average speech rate decreasing as information density increases across languages (Pellegrino et al., 2011), and locally, with local speech rates and prosodic prominence observed to vary with the predictability of the current segment, both for syllables (Aylett and Turk, 2004) and words (Bell et al., 2003).

A similar picture emerges when we look at timing effects between speakers in dialogue. First, speakers affect each other as regards the word- or segment-level timings discussed above: both speech rate and information density converge amongst interlocutors (Giles et al., 1991, give a summary), with some evidence that degree of convergence is related to high-level interpersonal factors such as the level of cooperation (Manson et al., 2013). Second, conversational participants are apparently experts in timing at the level of utterances or *turns* (segments during which one speaker holds the conversational floor). Sacks et al. (1974) show that turn-taking is far from random: the floor can be taken or surrendered at specific *transition relevance places*, and speakers and hearers are apparently aware of these and able to exploit them. Stivers et al. (2009) show that these abilities are cross-linguistic and cross-cultural: speakers and hearers manage the timing of these transitions to avoid overlap, and minimize silences; and experiments suggest that disruptions in natural interaction timings are noticed by infants as young as 3 months (Striano et al., 2006). Heldner et al. (2013) extend this to the more specific idea of *backchannel relevance spaces*, showing that even simple feedback vocalizations (e.g., “uh-huh”) are governed by constraints of appropriate timing.

Crucially, studies of turn-taking show that inter-speaker transition times are too short for this behavior to be reactive: if we waited for the end of the previous turn to react, we simply wouldn't have enough time to plan, select lexical items and begin to speak (requiring of the order of 600 ms) within the durations observed empirically (c. 200 ms). We must therefore predict the end (and content) of turns as we hear them, to begin our own response (see Levinson and Torreira, 2015; Levinson, 2016). Expectation is therefore key to turn-taking: EEG experiments show correlates of turn-end anticipation (Magyari et al., 2014), and models have been proposed based on syllable-timed oscillators (Wilson and Wilson, 2005). However, experiments suggest that this expectation is driven by factors at many levels. Grosjean and Hirt (1996) show that prosody helps listeners predict when a turn is going to end, although its utility depends on language and on position in the sentence. However, De Ruiter et al. (2006) asked participants to predict end-of-turn times with various manipulated versions of recorded speech: their predictions were accurate when hearing the original recordings, and when the intonation information was removed; but accuracy dropped when *only* intonation information was present and words could not be understood. Magyari and De Ruiter (2012) showed similar results when asking participants to predict the words remaining in a sentence. In machine classification tasks, Noguchi and Den (1998) and Ward and Tsukahara (2000), among others, show success in predicting backchannel points using prosody; but in a general turn-end detection task, Schlangen (2006) showed that combining acoustic, lexical and syntactic information improved accuracy, and Dethlefs et al. (2016) show that people's tolerance of speaker overlap depends on information density as well as syntactic completeness. While prosody contributes information, then, lexico-syntactic or higher levels must contribute as much if not more.

It is clear, then, that rhythmic structure pervades language, but that its perception and production are governed by expectation both within and between speakers—with this expectation based on information at a variety of levels. IDyOT theory proposes that this expectation is generated by the same general mechanism as that which affords musical meter perception, which is the topic of the next section.

### 3.5. Rhythm and meter in music

A distinction commonly made in the literature is that between musical meter and rhythm, although there is debate over the extent to which they can be treated independently (Cooper and Meyer, 1960; Benjamin, 1984; Hasty, 1997). London (2012, p. 4) defines rhythm as involving “patterns of duration that are phenomenally present in the music.” Duration here refers not to note lengths, but to the *inter-onset interval* (IOI) between successive notes. Rhythm therefore refers to the arrangement of events in time, and in that sense can be considered as something that exists in the world and is directly available to our sensory system.

Meter can be thought of as the grouping of perceived beats or pulses, simultaneously extracted from and projected on to a musical surface, into categories, which is typically expressed as the “regular alternation of strong and weak beats” (Lerdahl and Jackendoff, 1983, p. 12). London strongly situates meter as the perceptual counterpart to rhythm:

[M]eter involves our initial perception as well as subsequent anticipation of a series of beats that we abstract from the rhythmic surface of the music as it unfolds in time. In psychological terms, rhythm involves the structure of the temporal stimulus, while meter involves our perception and cognition of such stimuli. (London, 2012, p. 4)

The experience of meter can, therefore, be considered as a process of categorical perception, where the surface details of the temporal stimuli, such as the particular structure of the rhythmic pattern, or any expressive performance timing, are perceived with reference to a hierarchical organization of regular beats. The sensation of meter is induced from a stimulus in conjunction with both innate and learned responses to periodic or quasi-periodic stimuli.

Extending the notion of categorical perception, London (2012) argues that meter is a form of sensorimotor entrainment, that is a “coupled oscillation or resonance,” afforded by the temporal invariances commonly present in musical structure. For listeners, this is one mechanism by which attentional resources can be directed toward predicted salient timepoints to efficiently process complex auditory stimuli. For musicians, and indeed any form of movement associated with musical stimuli, entrainment is necessary for the co-ordination of physical action.

London (2012) provides empirical support for his theory of meter as entrainment from recent advances in neuroscience, which shed light on the underlying biological mechanism of rhythmic perception. Neuroimaging studies have discovered patterns of neuronal activity sympathetic with metrical entrainment, providing convincing evidence that metrical perception is both stimulus driven and endogenous. Differing EEG responses to trains of identical pulses are reported by Brochard et al. (2003) and Schaefer et al. (2011) as evidence for subjective metricization. Snyder and Large (2005) and Iversen et al. (2009) both present findings that lend support to endogenous neural responses correlating with accents that are only loosely coupled with external stimuli, and in the later study it is also demonstrated that the priming of an endogenous meter has a predictable effect on subsequent auditory responses. Nozaradan et al. (2011) present evidence of measurable neural entrainment to perceived and imagined meter.

The degree to which listeners can induce a sense of meter from a rhythmic surface has also been shown to strongly affect ability in reliably processing rhythmic information (Grube and Griffiths, 2009). Where a stronger sense of meter is induced, participants could more accurately detect rhythmic deviations. In the same experiment, the authors also provided evidence suggesting the importance of closure at the endings of rhythmic stimuli in order for listeners to report a stronger sense of perceived rhythmicality. Open endings were shown to leave listeners feeling uncertain about the structure of rhythmic stimuli, demonstrating how the ends of sequences can influence the perception of the whole.

Composers have long exploited our capacity to maintain a metrical context (i.e., our capacity for sustained voluntary entrainment), which is possible even in the presence of conflicting musical stimuli. Syncopation is the intentional rhythmic articulation of less salient metrical timepoints, which in itself is evidence for our strong tendency for entrainment, since if we could not independently maintain a sense of meter the concept of “off-beat” would be meaningless. The notion of a continuous oscillation in attentional energy provides an account, importantly one with an empirically grounded underlying mechanism, of the commonly held view that meter concerns regular patterns of strong and weak beats.

### 3.6. Affective responses to expectation in timing

Huron (2006) argues that prediction, experienced as expectation, is a driver of musical affect. Huron proposes that the feeling of *uncertainty*, which corresponds with entropy in a predictive distribution (Hansen and Pearce, 2014), makes a substantive contribution to the aesthetic of music: changes in tension due to changes in uncertainty resolving into expected certainty, or denial of expectation, is sometimes called the “ebb and flow” of music. Empirical evidence of this relationship is supplied by Egermann et al. (2013): correspondence was found, by direct and indirect response, between affective change and change in information content as predicted by Pearce's *Information Dynamics of Music* (IDyOM) model (Pearce, 2005; Pearce and Wiggins, 2012).

However, anticipation of what is coming next (followed by the outcome and its concomitant affect) is only one aspect of this response. Another key aspect is the entrainment that allows groups of humans to perform music together, in perfect but flexible, consistent time, in ways which have never been demonstrated in other species.

An open question is why the act of entraining should produce positive affect, as it does (Hove and Risen, 2009; Tarr et al., 2015). One possible answer is that, because cognitive entrainment is necessary for efficient speech communication (see Section 3.4), mutations that select for entraining capacity, and also for exercising of that capacity are favored. Thus, a capacity which is, presumably, grounded in fundamental cyclic behaviors such as locomotion (Fitch, 2012), might be exapted to support communication through speech, but also social bonding through shared musical activity. Since speech and social bonding are interlinked, and social bonding is crucial to human survival in the wild, one can postulate a tight feedback loop between these various factors, leading to the advanced capacity for musical and speech rhythm in modern humans. This account places neither music nor language as the progenitor: it would be the basis of an evolutionary theory in which they develop in parallel from a common root, possibly through shared mechanisms and/or resources.

There remains something of a lacuna in the literature on musical affect, with respect to specific small-scale deviations, as in groove. It is to be hoped that a model like IDyOT will render hypothesis formation in this area more readily achievable, and thence empirical study may be enabled. However, in both speech and language, affect is manipulated, intentionally or otherwise, by both time and pitch—as in the frustrating denial of expectation by a speaker who pauses too much, or by a performer whose timing is poor. Kant (1952) proposes a theory of *incongruity* for positive affective response in humor, and something similar to this may apply here; however, we reserve this discussion for future work.

Here, what is important is that the expectations generated in time form a predictable, if locally irregular, structure, and small variations in that structure are desirable, giving rise to affective responses such as “feeling the groove” in music (Witek et al., 2014) and “pause for emphasis” in language (Cahn, 1990). This entails a representation in which a norm (the standard beat, isochronous or otherwise) is directly implied, but in which variation may be quantified so that further prediction and associated affect may be modeled. Such a representation is the subject of the next section.

### 3.7. A conceptual space of rhythm and meter

#### 3.7.1. The theory of conceptual spaces

Gärdenfors (2000) proposes a theory of conceptual spaces as a geometric form of representation, situated between sub-symbolic and symbolic representation. The theory proposes that concepts—entirely mental entities—can be represented using sets of dimensions with defined geometrical, topological or ordinal properties. The formalism is based on *betweenness*, from which a notion of conceptual similarity is derived.

Gardenfors' theory begins with an atomic but general notion of *betweenness*, in whose terms is defined *similarity*, represented as (not necessarily Euclidean) distance. This allows models of cognitive behaviors to apply geometrical reasoning to represent, manipulate and reason about concepts. Similarity is measured along *quality dimensions*, which “correspond to the different ways stimuli are judged to be similar or different” (Gärdenfors, 2000, p. 6). An archetypal example is a color space with the dimensions hue, saturation, and brightness. Each quality dimension has a particular geometrical structure. For example, hue is circular, whereas brightness and saturation correspond with measured points along finite linear scales. Identifying the characteristics of a dimension allow meaningful relationships between points to be derived; it is important to note that the values on a dimension need not be numbers—though how an appropriate algebra is then defined is not discussed.

Quality dimensions may be grouped into *domains*, sets of *integral* (as opposed to *separable*) dimensions, meaning that every dimension must take a value to be well formed. Thus, hue, saturation, and brightness in the above color model form a single domain. Each domain has a distance measure, which may be a true metric, or otherwise, such as a measure based on an ordinal relationship or the length of a path between vertices in a graph. Thence, Gärdenfors' definition of a conceptual space is “a collection of one or more domains” (Gärdenfors, 2000, p. 26). For example, a conceptual space of elementary colored shapes could be a space comprising the above domain of color and a domain representing the perceptually salient features of a given set of shapes.

Since the quality dimensions originate in betweenness, similarity is directly related to (not necessarily Euclidean) proximity. Such spatial representations naturally afford reasoning in terms of spatial regions. For example, in the domain of color, a region corresponds with the concept red. Boundaries can be adaptive, providing the formalism with an elegant means of assimilating new knowledge, and the space itself can be subject to geometrical transformation, such as scaling of constituent dimensions, modeling shifts in salience. For purely numerical dimensions, Gärdenfors (2000, pp. 24–26) tentatively suggests Euclidean distance for similarity in integral dimensions, and the city-block metric for separable dimensions.

#### 3.7.2. A geometrical formalization of meter and rhythm

Forth (2012) formalized London's theory of meter (London, 2012, Section 3.5), seeking quality dimensions to express all the ways in which metrical structure may be variable in perception. Forth (2012) specifies two conceptual space representations of metrical structure, denoted meter-p and meter-s, to enable geometrical reasoning over metrical-rhythmic concepts. The simpler space, meter-p, represents the periodic components of well-formed hierarchical structures that correspond with metric entrainment. It can accommodate all theoretically possible forms of metrical structure, while entrainment itself is bounded by fundamental psychological and physiological constraints (London, 2012). The principal affordance of the geometry is direct computation of similarity between musical rhythms with respect to a metrical interpretation. In a genre classification task, exemplars of a range of dance music styles were projected as points in each space. Applying simple nearest-neighbor clustering over the points in each space, classification accuracy of 76% and 81% was achieved for meter-p and meter-s respectively, compared to the naïve classification baseline of 22%.

The overall general spaces are quite high-dimensional, but current thinking is that any individual actually uses a subspace, attuned to their enculturation. Thus, someone enculturated purely in Western rock would not have in their conceptual space the dimensions required to capture, say, the Yoruba timeline, explaining why even accomplished Western musicians must learn to relate to such non-Western metrical structures. The dimensionality of the spaces depends, also, on the number of metrical levels instantiated in the overall metrical structure. Therefore, musically less structured rhythms inhabit a lower dimensional space; and, conversely, each space may be extended by the addition of new dimensions corresponding to higher-level groupings or lower-level beat subdivisions.

An important aspect of this representation is its ability to abstract metrical structure from the tempo and expressive variation of individual performances. While it is possible to instantiate the representation to the point at which specific real times are included, and thus actual performances are represented, these times may be abstracted out. In this case, a point in the abstracted subspace represents a schematic, regularized version, which may capture multiple performances of a given rhythm: and so the region that the individual performances inhabit constitutes a concept under Gärdenfors' notion of convexity. The geometry of the space then allows us to distinguish groove, inconsistent timing errors, and tempo change because of their different statistical properties: the first is a tightly defined point slightly away from the regularized rhythm, the second is a cloud around a regularized rhythm, and the third is a monotonic trajectory around the regularized rhythm. These diagnostic properties both provide support for the hypothetical representation and afford a useful facility in the wider theory proposed below.

## 4. IDyOT: the information dynamics of thinking

### 4.1. A predictive cognitive architecture

We now outline the IDyOT architecture. The aim of the current section is to explain enough detail to allow the reader to follow our account of the timing aspects. Further explanation is given by Wiggins (2012b) and Wiggins and Forth (2015).

**Figure 1:**
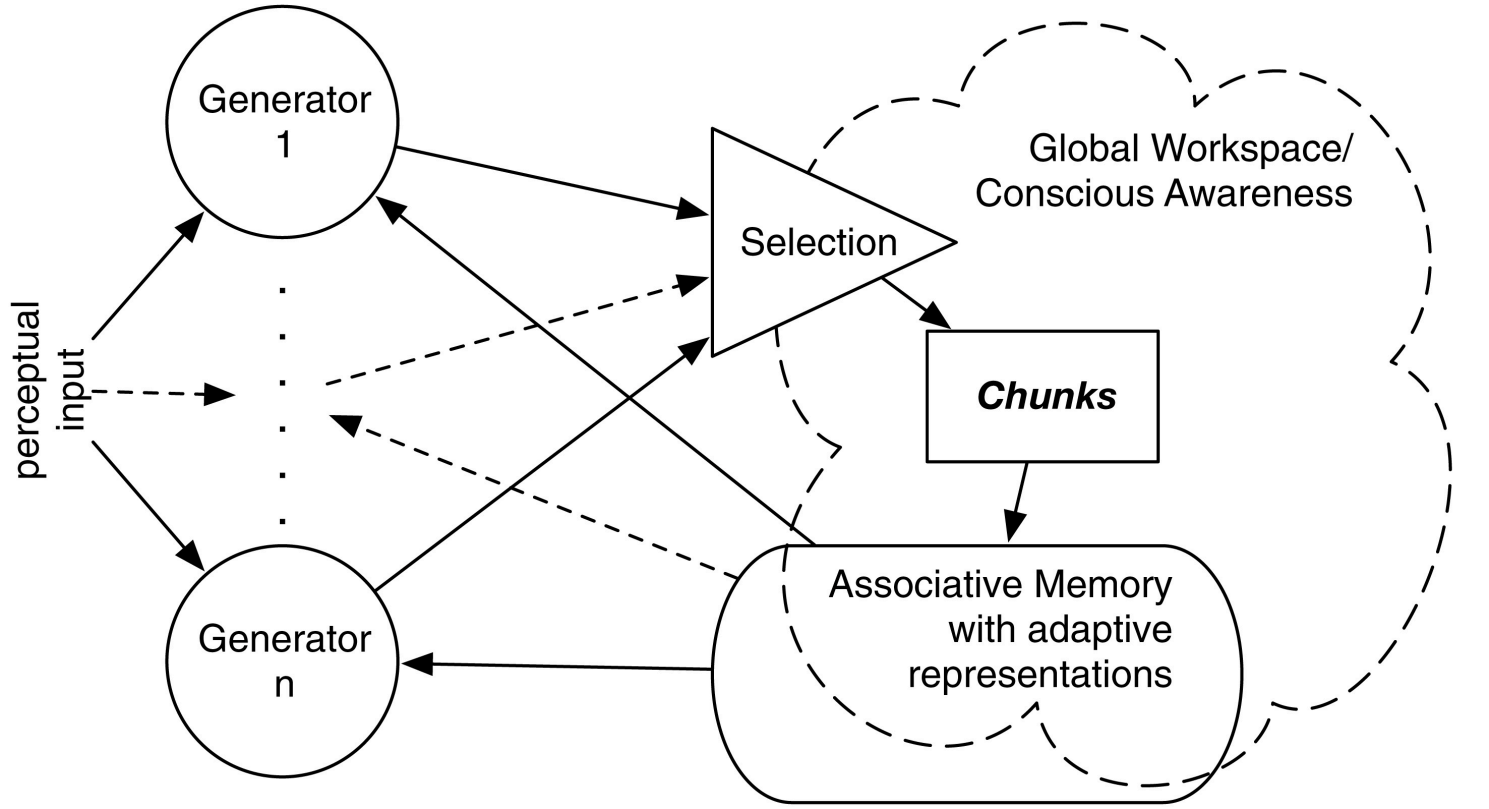
**Overview of the IDyOT architecture**. Generators synchronized to perceptual input sample, given previously buffered perceptual input (if any), from a first-order, multidimensional Markov model to predict the next symbol in sequence, which is matched with the input. Predicted symbols that match are buffered by each generator until it is selected on grounds of its information profile. The selected generator then flushes its buffer into the Global Workspace, which is the sum of the structured hierarchical memory and a detector that searches for salient information, shown as “conscious awareness” here. This allows the resulting chunk of sequence to be stored in the memory, to become part of the statistical model and thence to be used subsequently.

IDyOT implements Baars' Global Workspace Theory (GWT; Baars, 1988), affording a computational model of hypothetical cognitive architecture. GWT is primarily intended to account for conscious experience, and that is relevant to some aspects of IDyOT theory. However, it is the underlying mechanism that is of interest here, in our references to both theories. A number of *generators* sample from a complex statistical model of sequences, performing Markovian prediction from context (Wiggins and Forth, 2015). Conceptually, each generator maintains a buffer of perceptual input which may include mis-perceptions and alternative perceptions due to the possibility of multiple predictions matching ambiguous or noisy input, expressed as symbols, whose origin is explained below. Each buffer serves as a context for prediction of the next (as yet unreceived) symbol; predictions are expressed as distributions over the alphabet used to express the input. A buffered sequence is flushed into the Global Workspace when it meets a chunking criterion as described below. Figure 1 gives an overview; see Wiggins (2012b), Wiggins and Forth (2015) for more detail.

IDyOT maintains a cognitive cycle that predicts what is expected next, from a statistical model, expressed in terms of self-generated symbols that are given semantics by perceptual experience. IDyOT is focused on sequence, and this is in part due to the musical focus of its ancestor, IDyOM (Information Dynamics of Music: Pearce, 2005; Pearce and Wiggins, 2012). IDyOM models human predictions of *what* will happen in an auditory sequence, and takes account of information about musical time in making its predictions. It is the most successful model of musical pitch expectation in the literature (Pearce and Wiggins, 2006), but it cannot predict *when* the next event will fall in a statistically defensible way, and it is a static model, operating over a body of data viewed as a fixed corpus: it has no interaction with the world; it has no real-time element. The focus of this paper is to extend the IDyOT model with timing, to show how it accounts for musical meter, potentially in real time.

**Figure 2:**
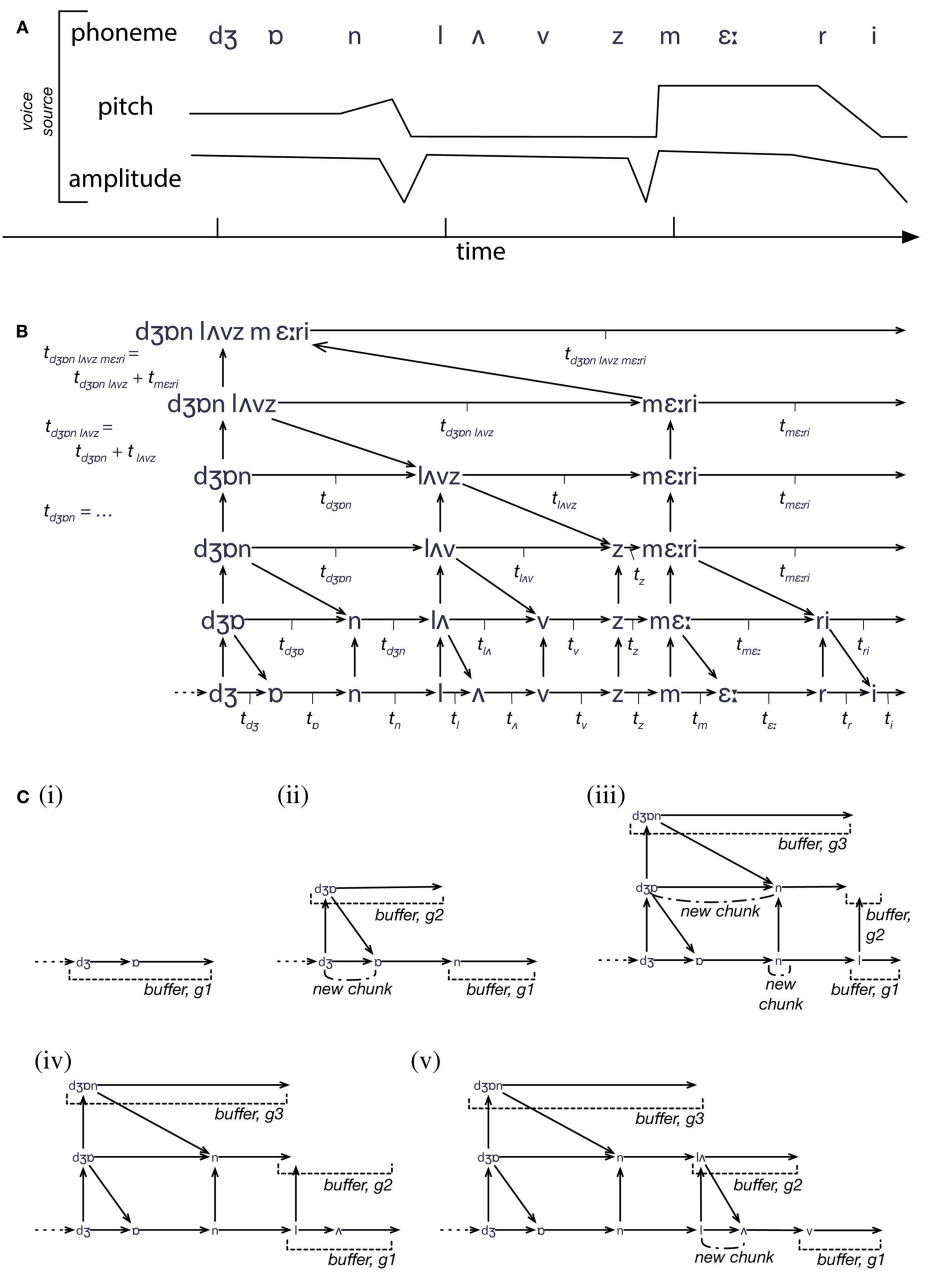
**An illustration of the process of IDyOT structure building**. **(A)** The input to IDyOT is a sequence of values, with three different features (thought of as *viewpoints* after Conklin and Witten, 1995). In reality, the voice input would be an audio signal, but for the purposes of example, we start at the (abstract and approximate) phoneme level. The sentence perceived here is “John loves Mary.” **(B)** The structure eventually developed by IDyOT, showing the hierarchical model created by information-theroetic chunking, and the individual times associated with each chunk (as used in Figure 4) and the higher-level symbol that labels it. **(C)** Five steps in the construction of the memory structure shown in **(B)**. A generator is associated with each level of each viewpoint, and with each alternative reading of the structure (though ambiguity is not shown here: see Wiggins and Forth, 2015, for details). Rather than move data around, new input, once matched perceptually, is added directly to the memory, which serves as the substrate of the Global Workspace. As each chunk is constructed, there is a peak of information content, which constitutes attentional energy in the system. Thus, as larger chunks are produced up the hierarchy, larger segments of text (and of the meanings with which they are associated) enter the Workspace; this accods with the “spotlight” analogy of Wiggins (2012b).

Figure 1 illustrates the cyclic (and hence dynamical) nature of the IDyOT model. The generators sample from statistical memory, synchronized by its own expectations of the perceptual input, if some exists, that it receives. If there is no input, the generators freewheel (Fink et al., 2009; Wiggins and Bhattacharya, 2014), conditioned only by prior context, and this is where creativity is admitted. In the current paper, we focus on the perceptual input and synchronization. Perceptual input is matched against generators' predictions, and where a match leads to a larger increase in uncertainty than other current matches, the corresponding generator's buffer is emptied into the Global Workspace, which is in fact IDyOT's memory adormed with buffers along its leading edge (Figure 2). The previous buffer now forms a perceptual chunk, linked in sequence with the previous chunk. The model entails that at least some generators must be working in all perceptual modalities at all times, including sensory ones; otherwise nothing would be predicting for new input in a given modality to match against. The process of structure generation is explained in Figure 2.

As in parsing by competitive chunking (e.g., Perruchet and Vinter, 1998; Servan-Schreiber and Anderson, 1990), IDyOT's chunking process breaks percept sequences into statistically coherent groups, which tend to correspond with structurally coherent sub-phrases, though not necessarily with traditional linguistic categories. Chunking is the basic process by which IDyOT manages its information, by analogy with human perceptual chunking (Gobet et al., 2001). Once a chunk has entered the Global Workspace, it is added to the memory and becomes available to the generators for prediction. This generates a positive feedback loop in which the chunks inform the statistical model that in turn causes chunking, reinforcing the model.

Each chunk, having been recorded, is associated with a symbol in the next-higher-level of the model, which in turn adds to the overall predictive model, and each higher level is subject to chunking. Each symbol corresponds with a point in a conceptual space associated with its own layer, and each such point corresponds with a region or subspace of the conceptual space (Gärdenfors, 2000) of the layer below, defined by the lower-level symbols in the chunk. Thus, two representations grow in parallel: the first symbolic and explicitly sequential, driven by data, providing evidence from which the second is derived; and the second geometrical, mostly continuous, and relational higher layer, providing semantics for the symbols of the first.

For symbol tethering (Sloman and Chappell, 2005), very low-level conceptual spaces are *a priori* defined by the nature of their sensory input (inspired by human biology); higher-level ones are inferred from the lower levels using the information in the sequential model. The exact nature of the conceptual spaces involved is an interesting future research area. A measure of similarity, borrowed from conceptual space theory (Gärdenfors, 2000), allows structures to be grouped together in categories, giving them semantics in terms of mutual interrelation at each layer, and tethering to the level below, eventually bottoming out in actual percepts. Using this, a consolidation phase allows membership of categories to be optimized, by local adjustment, in terms of the predictive accuracy of the overall model. Theoretically, the layering of models and its associated abstraction into categories can proceed arbitrary far up the constructed hierarchy. For clarity here, we restrict our example to the number of layers necessary to describe simple musical rhythms.

**Figure 3:**
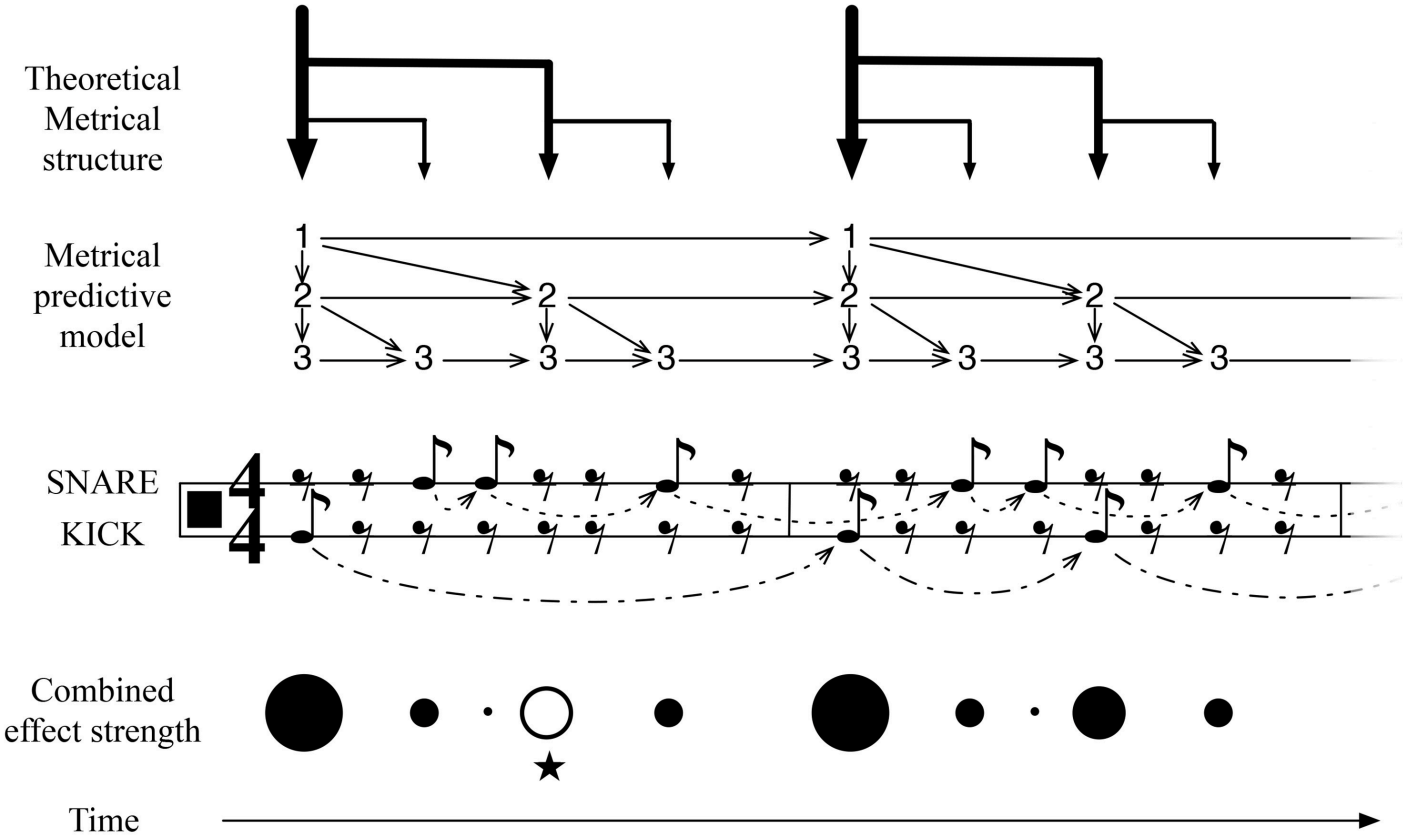
**An illustration of IDyOT learned representation of meter, for simple duple time, and its application to an experienced simple rock rhythm**. This structure is learned from exposure to meters of this form, optimized by IDyOT's memory consolidation process. It is compared here with the conventional music-theoretic ${}_{4}^{4}$ meter: the notation is restricted to quaver (eighth-note) values, to avoid giving the impression of *a priori* metrical structure. The score fragment denotes an experienced rock rhythm. The learned model of meter implies stronger or weaker anticipation over the relevant time periods, the whole being parameterized by the current basic unit. The model of the current rhythm (also learned, in the short term) also adds predictions. The rhythm shown here is a simple rock beat, which is interesting because of the anticipated third beat in the measure, marked with ⋆ here. This is an example where an expectation of the background model is denied and the resulting feeling of the missing beat is predicted.

In summary, IDyOT's memory consists of multiple structures, of which those in Figure 3 are simplified examples, in parallel, tied together by observed co-occurrences of feature values expressed in multidimensional perceptual input sequences. The whole constitutes a Bayesian Network, stratified in layers determined by the chunking process, and constrained to predict only to the subsequent symbol at each level and in each modality. Note, however, that the subsequent symbol may represent something arbitrarily far in the perceptual future, because higher-level, more abstract models predict in parallel with, and conditioned by, more concrete ones, and each higher-level symbol will subtend more than one lower level symbol. From this model, IDyOT's generators make predictions and their outputs are selected on the basis of probabilistic matching with input. The differences between generator outputs is caused either by their predicting from different parts of the memory structure (e.g., at different levels in the hierarchy), or from stochastic choices licensed by the distributions with which they work.

### 4.2. Rhythm and timing expectation in IDyOT

Figure 3 illustrates the pattern of structures that is learned as a result of exposure to a broad range of ${}_{4}^{4}$ rhythms. (The model will, of course, be much more complicated than this in general, because other meters will be represented in the same network.) The binary structure results because of a combination of musical practice, in which event occurrences on metrically strong pulses are more frequent than on weak ones, and because a balanced tree representation of the structures is more information-efficient as a representation than other kinds of representation. Thus, the properties of the data to which IDyOT is exposed conspire with its information-based criteria to provide a theoretical account for the development of meter in humans. Any rhythm that IDyOT encounters is processed in context of this background model. The figure illustrates how the temporal expectations of the different metrical levels fit together to produce weaker and stronger temporal expectations at different stages in the meter, with the perceived effect shown in the *Metrical Structure* and *Combined effect strength* illustrations.

The IDyOT generators make predictions of what will be perceived next, expressed as distributions over the relevant alphabet. Each generator also makes a prediction of when the relevant symbol will appear. Because more predictors from different levels predict (what would musicologically be) strong beats, the prediction at these points is correspondingly stronger, and, in terms of qualia, this affords the experience of metrical, hierarchical rhythm.

Section 3.7.2 outlines how the conceptual space of meter and rhythm proposed by Forth (2012) affords generalization away from the details of particular performances, to corresponding patterns of entrainment, and allows the analysis of variation in terms of its geometrical properties. Once such a space is established, new time intervals can be represented within it, and thence abstraction away from time interval to tempo becomes a straightforward projection operation on the space, rather than a matter of timing from the raw data alone, which would be difficult to handle without the prior knowledge encoded in the metrical model.

Importantly, the mechanism is required for effective linguistic communication with multiple individuals, who may have variation in speed or in regularity in their own speech, and who will certainly vary in speech speed from one to another; and for combining expectations driven by information at multiple levels, to allow accurate anticipation of lexical timing and sentence or turn-end timing simultaneously. Exactly the same hierarchical process can apply in both modalities.

Thus, IDyOT affords a method by which *sequences* of time periods may be derived from a base level of measured temporal units, which allows the construction of the metrical space from *tabula rasa*. Exposure to sufficient metrical data will cause the construction of hierarchical representations of meter, the hierarchies summing the durations of their subtended sequences, summarizing the rhythms in the data, as illustrated in Figure 3. The relationship between the basic unit, and the structures composed upon it by chunking, may be expressed by locating a rhythm as a point in the conceptual space defined in Section 3.7. Because the perceptual tendency is to integrate all concurrent rhythmic input, even when it is not obviously coherent, into a percept of one single rhythm (as in polyrhythms) the entire rhythmic structure that is audible at any point in time may be represented as exactly one point—or, if it is sufficiently uncoordinated as not to be perceptible as a rhythm, then as no point at all. Thus, the entire IDyOT Global Workspace resonates with the resultant temporal beat of its input, or descends into confusion when multiple conflicting rhythmic inputs are present.

The abstract, static representation afforded by the conceptual space, however, does not account for the on-going, dynamic percept of rhythmic beat: rather, it provides the parameters that configure it. In IDyOT, the on-going experience is accounted for, instead, by the predictive anticipation of the generators that use the memory at any point in a perceptual sequence to generate expectations. Consider a regular, Western rock beat, as illustrated in Figure 3, as processed by an IDyOT with extensive exposure to this kind of relatively foursquare rhythm.

First, we discuss predictions at the metrical level. At this level, the predictions of the part of the model representing the current rhythm are mostly in line with those of the more general metrical predictions, and therefore the expectations are reinforce: evidence confirms the estimating of the basic unit, and the predictions can be correspondingly more certain. This corresponds with a human feeling the beat strongly. However, there is one place in this rhythm where a specific musical effect is noticeable, that does not accord with simple prediction: on the third beat of the first measure, a strongly expected beat is not present in the rhythm (marked with ⋆ in Figure 3). Affectively, this *loud rest* (London, 1993) lies in strong contrast to the second measure of the rhythm, where the expectation is fulfilled. This rhythm, therefore, creates its musical effect by subverting the metrical expectation of the listener, and IDyOT is able to predict this effect: unexpected occurrences draw attention, and thus the listener is kept interested in the beat.

### 4.3. A hypothetical mechanism underlying entrainment

Entrainment in IDyOT is a direct consequence of attentional dynamics. Following Nobre (2001), IDyOT embodies a multifaceted view of attention, in which there is no “unitary homuncular attention system” (Nobre, 2001, p. 1326). The understanding of attention becomes distributed activation in neural assemblies, not a single function of the brain.

The mechanism with which IDyOT makes predictions of time is the novel contribution of this paper. We consider temporal predictions to be generated by the same kind of statistical process that governs the prediction of other attributes, such as the likelihood of particular musical pitches or phonemes of speech. However, temporal predictions are integral to the behavior of the cognitive system itself, in time. Temporal predictions are hypothesized as drivers or regulators, coordinating, but also influenced by, the generation of predictive distributions in other domains, which collectively constitutes the generation of *expectations*. The interaction between generated expectations and sensory input leads to the construction of representations in memory, which in turn conditions subsequent expectations.

Measuring time necessitates the ability to relate distinct moments across time, and a mechanism by which the distance between such markers can be determined. Although the actual mechanism is the subject of much debate (for an overview see Hass and Durstewitz, 2014), we assume a neuronal representation of the passing of time to be available in the brain. moments in time can be related with respect to this underlying clock, and that the neural encoding forms the basis for the estimation of time intervals, which may be related to activation in brain areas such as the pre-supplementary motor area and frontal operculum (Coull, 2004).

Hypothesizing an intervallic representation of time underlying the cognitive processing of temporal information may appear obvious. However, considering the question of why and how this may be the case illustrates and supports our wider position regarding the importance of prediction and efficiency of representation in perception and cognition. Analogous to the derivation of intervallic representations of pitch from absolute representations of pitch, an intervallic representation of time is more compact in terms of both alphabet size and resulting statistical model than a monotonic time-line. Furthermore, intervallic representations are invariant under translation, directly affording comparison, forming the basis for the identification of higher-level structure. We conjecture that the same mechanisms of chunking and representation learning, previously described as the core mechanisms underlying the processing of symbol sequences within the IDyOT cognitive architecture are directly applicable to the modeling of time, and in turn, underlie the real-time temporal dynamics of the cognitive system.

Multiple independent IDyOT generators continuously predict sensory input, at each level of the metrical hierarchy induced by the chunking process. There must be a sufficient number, making predictions at sufficient frequency, to be useful to the organism in any given situation, subject to the constraint of available cognitive resources. In the auditory domain, we take the lower bound of 20*ms* (the approximate minimum IOI at which listeners can reliably discern the correct ordering of two successive onsets: Hirsh, 1959), to determine the highest frequency at which the architecture must run—but note that this could result from generators running at this frequency, or from sufficiently many generators running more slowly, but coordinated to support this temporal resolution.

IDyOT generators exhibit weakly coupled behavior, because they infer their timings from the single hierarchical memory; however, no direct coupling mechanism is assumed between individual generators. Following the global workspace theory, we hypothesize that coupling behavior emerges as the phenomenal experience of meter via the role played in the architecture by the global workspace itself, through which all communication between generators is mediated.

It is parsimonious to argue that temporal expectations, in whatever modality, are generated in accordance with general predictive principles, which are sensitive to the statistical regularities, or invariances, of sensory input. The finite resources of cognition act as a global constraint on temporal structure, which in the limit tend toward maximizing efficiency. Therefore, we argue that the same kind of predictive temporal dynamics exists in both music and language, following the temporal structure of intentional and communicative behavior. In both cases, time is used to optimize attention and maximize communicative potential. In both cases the features of the stimuli condition temporal prediction, which in turn drive the prediction of these features in time.

Thus, conceptual space representations are learned because they are efficient, and they are constrained by embodiment, and therefore take a common form across a species, but are variable across culture. Thence, we hypothesize that the mechanism underlying entrainment is a process of modeling observable patterns, which may (in a natural organism) be associated with the cause of the patterns, and thus given meaning.

**Figure 4:**
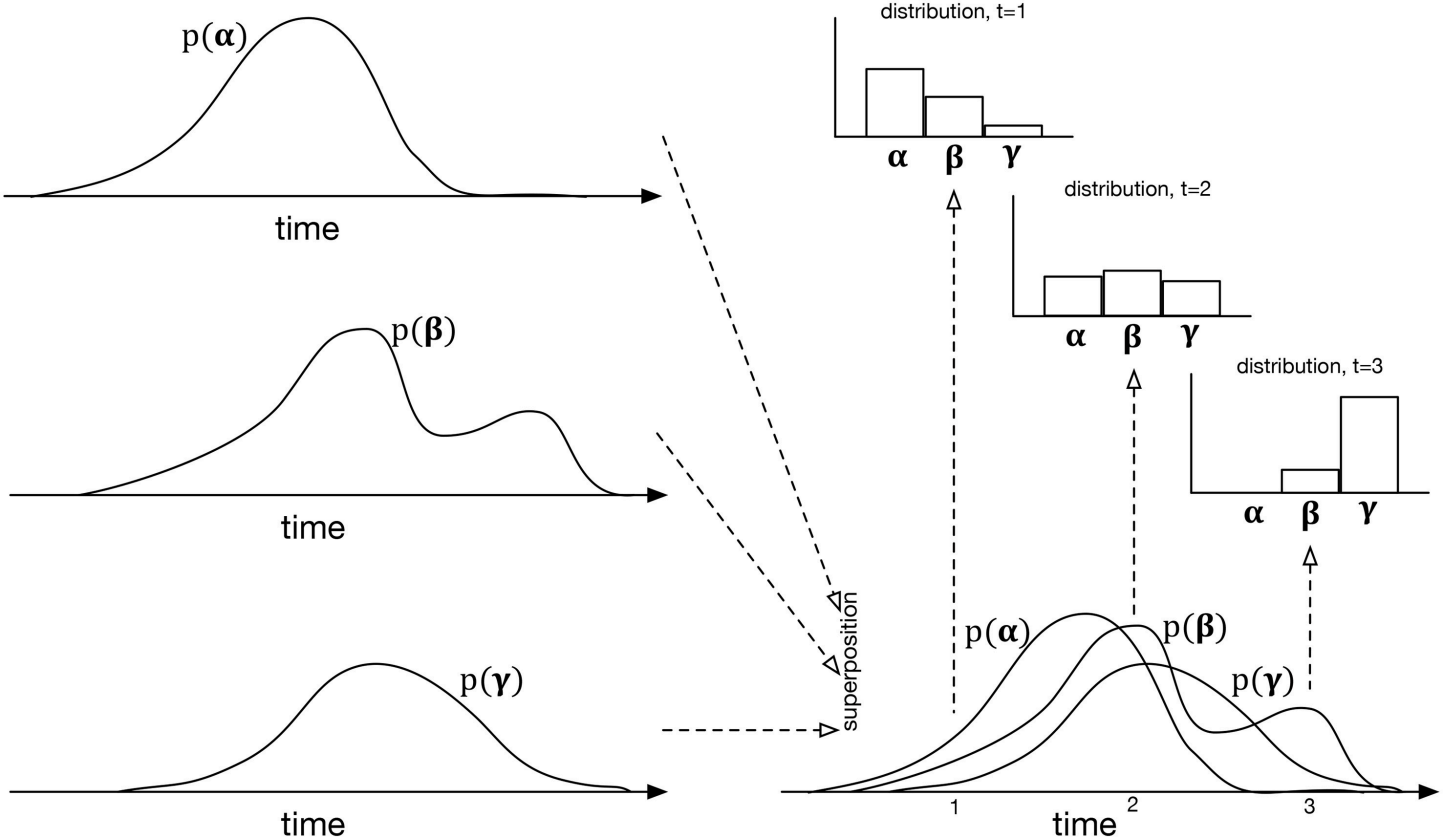
**The process of generating IDyOT dynamic distributions over an imaginary alphabet of three symbols, α, β, and γ**. The left side of the figure shows the time distribution for each symbol as generated from observed experience. These distributions may be notionally superposed, giving the bottom right diagram—however, note that this is for the purposes of illustration only: it is not intended to represent a combined distribution of any kind. Instead, given the superposition of temporal distributions, instantaneous ones may be generated, cutting “across” the temporal ones, the examples shown here being at *t* = 1, 2, 3. The resulting model is similar to a Markov renewal process (Ross, 1992), but extends the idea to hierarchical structure.

The specific mechanism proposed is an extension of the event-by-event prediction used in extant statistical models of music and language. As each event is detected, the next one is predicted, the prediction being expressed as a distribution over the symbols of the dimension being predicted. In IDyOT, differently, this distribution changes with time, time being substantially more granular than the inter-event interval. It can be calculated as follows. Instead of merely determining the observed likelihood of each of the possible symbols in context, IDyOT treats each piece of evidence differently, counting not only the symbols, but also the expected time of occurrence. The result may be viewed as an overlay of distributions in time, one for each symbol, with the overall distribution across the alphabet at any point calculated by looking up the value of each symbol at that point. This is illustrated in Figure 4; it affords one of the means of testing the IDyOT model, laid out in the next section.

To summarize: in IDyOT, the experience of pulse, defined by Fitch (2013) as a primary cognitive construct, emerges as an epiphenomenon of our more general notion of entrainment: it results from the superposition of multiple, regular strong expectations. Importantly, this theory explains how pulse can be imagined, rather than being a response elicited by actual sound, and how the intrinsic experience of pulse can continue beyond an audio stimulus. IDyOT's mechanism also accounts for loud rests (see Figure 3) and other effects such as the jolt experienced by listeners enculturated into simple Western rhythms when presented with simple non-isochronous time signatures such as ${}_{8}^{7}$. Perhaps most important, it explains how untrained children from middle Eastern cultures can clap easily along to rhythms that advanced Western musicians sometimes find challenging: because the rhythms are learned, and the learned model affords the entrainment, not some simple oscillatory mechanism.

Thus, entrainment in IDyOT is a more general concept: it emerges epiphenomenally from hierarchical time prediction over sequential structures. The strength of predictions is determined by memorized hierarchical information, leading to the multiple different strengths of expectation required to explain the experienced complexity of rhythm in both music and language, from simple pulse up to the extreme rhythmic complexity found in Arabic, Indian and African musics, and the complexity of rhythm in language from everyday argot to the most carefully performed poetry or rap.

## 5. Methodology: studying evolution through computational simulation

A perennial problem for evolutionary accounts of biological development is that of distinguishing them from Just-So Stories (Kipling, 1993), because they are untestable. Here, we propose a methodology in which computational models of cognitive process afford a means of testing hypotheses about evolutionary development. While it is clear that *in silico* simulation is not the same as running *in vivo* experiments over evolutionary time, it can help to supply evidence for argument, if it is done rigorously.

To see this, one must understand that the computational model in question is not merely a predictor from data. That is, it is not an attempt to neutrally machine-learn structure in data and classify on that basis, or to search for arbitrary correlations. Rather, it is in its own right an overarching theory about the *functional process* of mind, which may be decomposed into several related aspects, one of which (timing) is the current topic. Different aspects of the theory are testable in different ways, and only though a comprehensive programme of experimentation—first concentrating on individual aspects in isolation, then in combination—can a full understanding of the wider theory be established. From the current perspective, then, IDyOT is a theory; the aspect under scrutiny is its timing mechanism, and this drives our current hypothesis formation.

Given adequate evidence that the model is correct with respect to current biology, the evolutionary affordance of the approach becomes available. Once the model has been shown to be an acceptable predictor of empirical observations of the behaviors it claims to capture, its parameters may be changed so as to simulate the effect of changes known to have occurred in the relevant species over evolutionary time: e.g., size of organism and/or nervous system, availability of food, or other intrinsic or extrinsic factors.

To be clear: we do not claim that this methodology can directly simulate evolution in all its complexity, but we do claim that it can supply useful answers to carefully posed questions that have a bearing on the evolution of the aspects of present-day organisms that the model is shown to simulate.

## 6. Testable hypotheses

The IDyOT model affords more than one opportunity for exploration of human rhythmic behavior in language and music, and its evolution. First, the model must predict human behavior as currently observed, in both modalities. Because IDyOT is multidimensional, it is also possible in principle to study the effects of combining music with language, for example, in lyrics. Second, the model should be used to generate behavioral predictions, from which surprising examples can be extracted (Honing, 2006). These can then be tested against human behavior, further developing the model and adding to knowledge of that behavior. Thirdly, and more important in context of the current paper, parametric constraints may be placed on the model to explore hypothetical evolutionary pressures and help understand their effects. (Of course, this is only a valid approach if the model is demonstrated to be a good model of current humans.)

### 6.1. Metheds to validate IDyOT as a model of current cognition

There is a variety of empirical tests for music and language which may serve as validation of the IDyOT approach. For example, an IDyOT with greater hierarchical depth of processing, or more training examples, may be used to predict listeners with differing degrees of expertise or development, respectively; one hypothesis, for example, would be that there is a cutoff in terms of hierarchical memory depth beyond which language will be dysfunctional. In music, an IDyOT exposed to a large corpus may process musical structure at a higher level than an IDyOT exposed to a small corpus, in the same way that expert listeners tend to perceive music in terms of more semiotic structure; in this case, IDyOT's behavior could be compared with existing results on human behavior. In addition to modeling listeners with more or less musical training, IDyOT may be used to model the musical perception and expectations of listeners with different cultural backgrounds. Further, IDyOT may be used to model subjective metricization, to test whether an encultured IDyOT exhibits the same subjective metricization behavior as similarly encultured humans.

In contrast to specifically modeling listeners with divergent expectations (afforded from different cultural backgrounds or degrees of musical expertise), IDyOT may be used to simulate interaction between “average” listeners, or those of a comparable hypothetical listening background. Exposing trained IDyOTs to conversational dialogue should afford predictions of the timings associated with observed turn-taking and of human judgments in end-of-turn prediction experiments. Similarly, it should be able to generate expressive timing for synthesized speech that correlates with human affective response to timing deviations.

In similar vein, timing may be used to disambiguate language incrementally, as follows. Consider the following discourse fragments^1^:

1. There was a bank at the corner.
  /ðɛ: wɑz ə baŋk ət ðə ˈkɔ:nə/
2. There was a bang, catching my attention.
  /ðɛ: wɑz ə baŋ ˈkatʃɪŋ mʌɪ əˈtɛnʃən/

In fragment 1, the onset of the final /k/ phoneme of “bank” will appear somewhat earlier than the initial /k/ of “catching” in fragment 2, and thus the predicted meaning of the two sentences may changed at this very low level, as in the very eagerly predictive Cohort Theory (Marslen-Wilson, 1984) and its descendents. IDyOT theory predicts this and models the effect of the change in time explicitly, as illustrated in Figure 4. Note, however, that, on balance, semantic implication is usually somewhat stronger in disambiguating, as discussed by Wiggins and Forth (2015).

In the domain of music, IDyOT may be run as a participant in a tapping synchronization study, with the hypothesis that human-human pairings are indistinguishable from human-computer or computer-computer pairings. This sort of experiment would not only confirm the accuracy of the underlying mechanisms of IDyOT, but demonstrate the validity of the model when scaled up to behavioral interaction. More generally, we would hope that other known effects such as the scaling of timing errors proportionally to duration magnitude would be an emergent property of IDyOT's processing of sensory input, or that IDyOT can model how temporal predictions are modulated by non-temporal factors such that surprise, attention, high-level expectation from top-down knowledge.

In addition to modeling production and synchronization, as in a tapping study, IDyOT may be used to simulate human *perceptual* characteristics, such as the perception of similarity. Hypotheses could examine the formation of the model's geometrical space and probabilistic scaling of dimensions by testing whether the high level patterns captured by IDyOT are reflective of schematic perception of rhythmic variations, or of generalization and classification of linguistic information.

Another avenue of research with regard to language would be to test anomalies in perception and/or in the signal itself. And because IDyOT theory proposes that attention is regulated by information contained within the signal, its predictions can be experimentally validated with methods such as EEG (e.g., ERP Mismatched Negativity—MMN—analyses) or eye-tracking measurements, as these techniques capture the real-time dynamics of information processing. In one such experiment, IDyOT should be able to reliably detect a deviant item within a repetitive sequence, and therefore should accurately predict MMN response in oddball paradigms (Näätänen et al., 2007), for example. Rather than predicting neural response to anomalies, one may also predict human cognition at the behavioral level, by exposing a trained IDyOT to garden-path sentences, as discussed previously, or to semantically equivalent sentences which vary in hierarchical periodic temporal structure. In this later case, one would test whether IDyOT produces temporal responses comparable to humans (e.g., who make different end-of-sentence predictions). And finally, rather than testing ambiguous or unexpected sentence endings, one may also expose a trained IDyOT to nonsense words, to see whether the model, like humans, creates perceptual chunks, perceptually imposing more regularity in time than exists in the signal.

In the music information retrieval literature (see www.ismir.net), there is significant interest in so-called “beat tracking”—the automated detection of beat in (mostly popular) music, for the purpose of finding similar music for listeners. This not unsuccessful literature (e.g., Dixon, 2001, 2007; Davies and Plumbley, 2007) affords a rich vein of models against which to compare IDyOT's entrainment mechanism. Similarly, psychological (Povel and Essens, 1985) and neuroscientific (Patel and Iversen, 2014) comparators exist.

### 6.2. Predicting behavior from IDyOT

Following Honing (2006), once a model has been validated, the researcher should push the model toward the extremes of its parameters, to discover unexpected predictions about human behavior. This is a valuable step in testing and exploring a model's performance, because surprising predictions (1) may inform us about hitherto unknown (or not well understood) human cognitive mechanisms, and (2) will further validate the model in a broader range of behavioral contexts, by pushing the boundaries of what is known, not simply modeling expected behavior.

### 6.3. Correspondence with neural function

Our methodology is to model cognitive function abstracted from its substrate. However, it is useful to consider cognitive predictions in context of their hypothetical neurophysiological implementation, even though they are separated from it.

The function of IDyOT, however abstract, entails memory representations that increase in size with time. These representations, though not literal recordings of sensory experience, are very high-dimensional, because they connect all aspects of all features of sensory input together, where correlated. Unless one admits mysticism or quantum theory at the physical level of the brain (which we do not), this very dense interconnectedness entails the availability of brain volume which is strongly supralinear with respect to time, because every neural assembly (Hebb, 1949) has to be connected to every other relevant neural assembly, across modalities, between senses, and so on. This, we claim, is a necessary requirement of the established ability for veridical memory: we could not remember detail of a sonata or soliloquy unless it were so at some level of abstraction (notes/chords and words, respectively). Various antidotes to this effect may be proposed. For example: the low level detail of the memory may be discarded in favor of more abstract representations; or the layering of structures may be restricted to a given number of layers; or the connections between correlated sensory features may be limited; and so on. This affords a rich plethora of detailed hypotheses that may be tested in relation to comparative brain size of extant species with various cognitive capacities. This, in its own right, may be expected to elucidate the quality of the model in respect to these capacities; subsequently, in careful comparison with similar extinct species, it may be possible to chart a path relating increase in brain size with the development of successively more advanced cognitive capacities.

For example, it is known that dogs can perceive, remember, and associate meaning with words (that is, sequences of phonemes). But there is no evidence that they can compose words into meaningful phrase interpretations; indeed, quite the contrary. Our model would produce this effect when limited to only a few layers of chunking above the audio: sequences of phonemes, such as “walkies” would be memorable, but longer composed phrases and sentences would not. On the other hand, our theory affords much deeper construction when more layers are allowed (Wiggins and Forth, 2015).

Given this evolutionary account, one can formulate experiments based on IDyOT's ability to learn sequential structures (such as language or music) in which dependent variables relate to cortex volume: for example, the depth of layering can be limited, or the alphabets of the various layers can be limited, or both. These restrictions would be expected to limit the ability of the system to learn, and thence to predict. This approach, in particular, allows us to distinguish IDyOT from models whose parameters (e.g., node number in neural networks) are less specifically related to the function of the theory.

### 6.4. Evolving IDyOTs

From a modeling perspective, evolution may be thought of as a long-term parameter search within the IDyOT architecture and processing framework. When multiple IDyOTs exist in a genetic system, evolving freely, some will discover parameterization that allows for more efficient, evolutionarily adaptive behavior than others. Studies could be constructed such that IDyOTs with different temporal-predictive capacities will compete to survive, while the parameters of well-synced models are passed on to future generations by simulated breeding. The algorithmic parameters and probabilistic weightings underlying predictive processing may be randomly varied across agents to see which variations yield the most adaptive IDyOTs. Then, again, those whose predictions facilitate accurate communication or behavior may pass their algorithmic idiosyncrasies on to their IDyOT children.

In particular, parameters such as depth of hierarchy and retention of detail in symbol creation can be varied, and their effect on the predictions of the system studied. The most interesting possibility here is modeling the evolution of the neocortex: in the style of Bown and Wiggins (2005), an evolutionary computation system may be set up that allows simulation of not only cognitive function, but also the behavior of populations. Thus, evolution may be simulated quite literally *in silico*, albeit at a functional level, and the relationship between biological affordances and effects studied in ways that are not accessible *in vivo*.

## 7. Summary

In this paper, we have presented a novel model of timing in a predictive cognitive architecture. We have described in some detail how the temporal predictions allow efficient processing of ambiguous and/or noisy perceptual signals, and we have related the mechanisms to both linguistic and musical rhythm. Finally, we have proposed methods by which the approach will be evaluated, which constitutes the future work of the IDyOT project.

## Author contributions

JF invented the conceptual space representation and wrote about it, and also proposed much of the testing section; KA underpinned the theory with empirical research from psychology and neuroscience, and also contributed a lot to the testing section; MP supplied the linguistic grounding; GW invented the core model and wrote the sections summarizing it, and those on timing, using illustrations suggested by MP, and neural implementation. The rest of the writing was a team effort.

### Conflict of interest statement

The authors declare that the research was conducted in the absence of any commercial or financial relationships that could be construed as a potential conflict of interest. The reviewer JL and the handling Editor declared their shared affiliation, and the handling Editor states that the process nevertheless met the standards of a fair and objective review.

## Footnotes

^1^This example is oversimplified from a phonologist's perspective, because the /aŋ/ in “bank” and “bang,” would in reality change subtly to reflect what is coming next, but it serves to make the point here.
